# Targeting proinflammatory cytokines ameliorates calcifying phenotype conversion of vascular progenitors under uremic conditions in vitro

**DOI:** 10.1038/s41598-018-30626-z

**Published:** 2018-08-14

**Authors:** Björn Hegner, Theres Schaub, Daniel Janke, Daniel Zickler, Claudia Lange, Matthias Girndt, Joachim Jankowski, Ralf Schindler, Duska Dragun

**Affiliations:** 1 Charité – Universitätsmedizin Berlin, corporate member of Freie Universität Berlin, Humboldt-Universität zu Berlin, and Berlin Institute of Health, Clinic for Nephrology and Intensive Care Medicine, Campus Virchow-Clinic, Berlin, Germany; 2 Berlin-Brandenburg School for Regenerative Therapies (BSRT), Berlin, Germany; 3 Center for Cardiovascular Research (CCR), Charité University Hospital, Berlin, Germany; 4 Charité – Universitätsmedizin Berlin, corporate member of Freie Universität Berlin, Humboldt-Universität zu Berlin, and Berlin Institute of Health, Institute for Chemistry and Biochemistry, Berlin, Germany; 5 Clinic for Stem Cell Transplantation, Department of Cell and Gene Therapy, University Medical Center Hamburg-Eppendorf, Hamburg, Germany; 6 Department of Internal Medicine II, Martin-Luther-University Halle-Wittenberg, Halle, Germany; 7 Charité – Universitätsmedizin Berlin, corporate member of Freie Universität Berlin, Humboldt-Universität zu Berlin, and Berlin Institute of Health, Clinic for Nephrology, Charité University Hospital Campus Benjamin Franklin, Berlin, Germany; 8 Present Address: Vivantes Ida Wolff Hospital for Geriatric Medicine, Berlin, Germany; 9 Present Address: Charité – Universitätsmedizin Berlin, corporate member of Freie Universität Berlin, Humboldt-Universität zu Berlin, and Berlin Institute of Health, Institute for Cell- and Neurobiology, Campus Mitte, Berlin, Germany; 10 Present Address: Institute for Molecular Cardiovascular Research, University Hospital RWTH, Aachen, Germany; 11 School for Cardiovascular Diseases, Maastricht University, Maastricht, The Netherlands

## Abstract

Severe vascular calcification develops almost invariably in chronic kidney patients posing a substantial risk to quality of life and survival. This unmet medical need demands identification of novel therapeutic modalities. We aimed to pinpoint components of the uremic microenvironment triggering differentiation of vascular progenitors to calcifying osteoblast-like cells. In an unbiased approach, assessing the individual potency of 63 uremic retention solutes to enhance calcific phenotype conversion of vascular progenitor cells, the pro-inflammatory cytokines IL-1β and TNF-α were identified as the strongest inducers followed by FGF-2, and PTH. Pharmacologic targeting of these molecules alone or in combination additively antagonized pro-calcifying properties of sera from uremic patients. Our findings stress the importance of pro-inflammatory cytokines above other characteristic components of the uremic microenvironment as key mediators of calcifying osteoblastic differentiation in vascular progenitors. Belonging to the group of “middle-sized molecules”, they are neither effectively removed by conventional dialysis nor influenced by established supportive therapies. Specific pharmacologic interventions or novel extracorporeal approaches may help preserve regenerative capacity and control vascular calcification due to uremic environment.

## Introduction

Cardiovascular morbidity increases substantially with declining renal function culminating in patients with end stage renal disease, one of the populations with the highest risk for cardiovascular death due to massively accelerated intimal and medial calcification of arteries^1^. This specific cohort does not respond adequately to treatments that effectively reduce the risk for arteriosclerotic complications in the general population such as statins^2^. In addition to the well-established traditional risk factors several nontraditional risk factors have been identified including chronic inflammation, disturbed calcium-phosphate homeostasis and resulting hyperparathyroidism. Although high serum phosphate levels characteristic for patients with chronic kidney disease (CKD) and calcium induced apoptotic death of vascular smooth muscle cells (VSMC) initiate and propagate vascular extracellular matrix mineralization^3^ therapeutic strategies aiming to correct disturbed metabolism of calcium and phosphate also fail to substantially improve cardiovascular outcomes^4^. This therapy refractory pro-arteriosclerotic state is a consequence of the unique uremic microenvironment comprised by a complex mixture of more than 100 known and yet unknown uremic retention solutes (URS) contributing to systemic and cellular malfunction^5–9^. Although a negative impact of URS on anti-inflammatory cellular surveillance has been demonstrated^10^, little is known about their individual impact. Numerous URS have been shown to increase calcification of VSMC but their relative contribution to vascular calcification is indefinite.

Uremic calcific arterio- and arteriolopathy^11^ evolve by an active cell-mediated process that resembles intramembranous and endochondral bone formation^12,13^. Phenotypic conversion of de-differentiated VSMC towards osteochondrocytic cells is a key biologic process. In addition, cells of the vascular maintenance system such as multipotent mesenchymal stroma cells (MSC) functioning as progenitors to VSMC^14^ also have a high capacity for osteoblastic transformation^15–17^ linked to arterial calcification^18^. As a consequence, endogenous vascular regeneration is severely impaired.

Goal of our study was to identify potential therapeutic targets for the restoration of regenerative capacity of vascular progenitors in uremia since regenerative approaches might be more successful when damage is already established as is the case in patients with progressive loss of kidney function reaching end-stage renal disease. We employed an unbiased approach to identify individual components of the uremic milieu with the strongest capacity for induction of osteoblastic cell transformation in vascular progenitors crucial for development of calcific arteriopathy. Following the recommendations of the European Uremic Toxin Work Group (EUTox)^8^ we screened 63 individual substances that accumulate in patients with end stage renal disease for their influence on calcification of MSC. Interleukin-1β (IL-1β), tumor necrosis factor-α (TNF-α), fibroblast growth factor-2 (FGF-2), and full-length parathyroid hormone (PTH1-84) were the most potent enhancers of MSC calcification in a pro-calcific milieu. We provide translational proof-of-concept that individual pharmacologic blockade of the identified pro-inflammatory cytokines and growth factors is capable to effectively attenuate uremic microenvironment induced calcification of MSC. These findings allow for the development of novel targeted therapeutic strategies against vascular calcification in patients with chronic renal failure.

## Results

### Individual potential of URS to enhance osteoblastic differentiation of vascular progenitor cells

**Figure 1:**
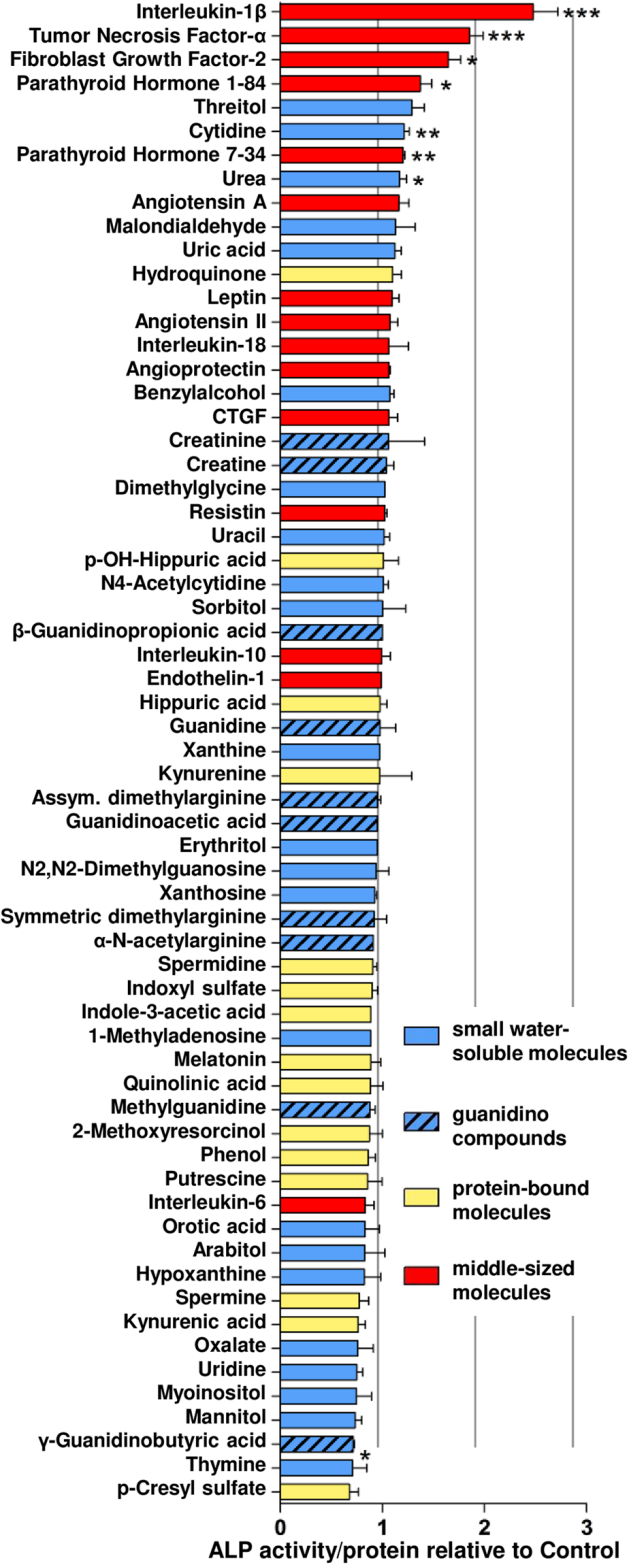
Screening of individual URS for promoting osteoblastic differentiation of MSC. ALP activity in MSC treated with 63 individual substances in osteoblast induction medium was normalized to protein content. Solvent control = 1.00. Means + SEM, n ≤ 10. *P < 0.05, **P < 0.01, ***P < 0.001.

Pluripotent undifferentiated MSC (Fig. S1) were exposed to a panel of individual URS for one week at the highest concentrations reported for dialysis patients (Table S1). Activity of alkaline phosphatase (ALP), a key osteoblast enzyme, served to quantify phenotypic conversion towards osteoblast like cells. Seven compounds increased ALP activity by more than 1.2-fold of control indicating enhanced osteoblastic MSC differentiation (Fig. 1). The pro-inflammatory cytokines IL-1β (2.48-fold of control) and TNF-α (1.85-fold) were the strongest inducers of MSC osteoblast differentiation followed by FGF-2 (1.65-fold) and PTH1-84 (1.37-fold).

### IL-1β, TNF-α, FGF-2, and PTH1-84 are potent inducers of MSC osteoblastic differentiation and calcification

**Figure 2:**
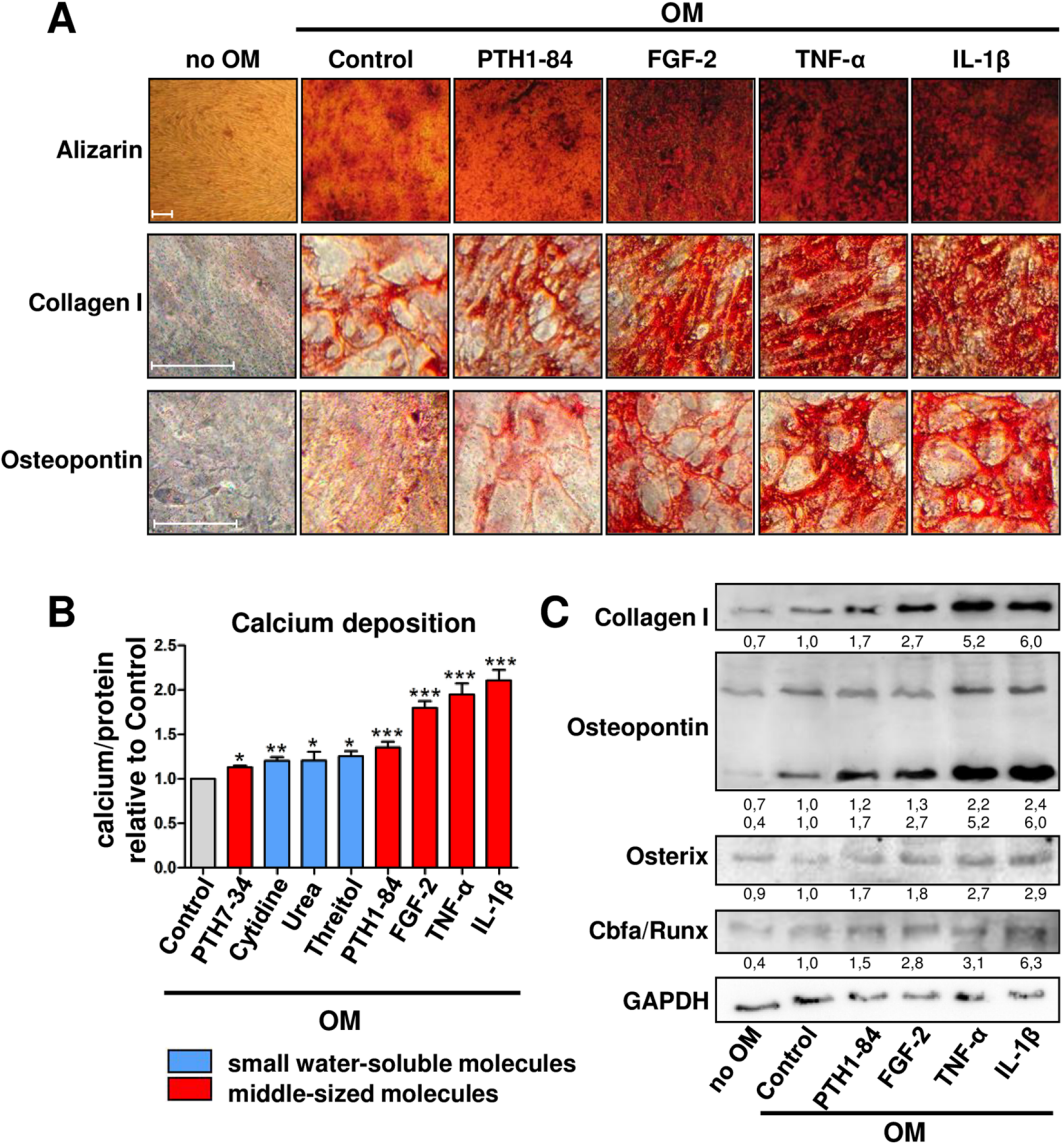
Osteoblastic differentiation of MSC induced by URS that increased ALP activity by more than 1.2-fold. MSC were cultured in OM. (**A**) Alizarin staining for extracellular calcium deposition and immunocytochemistry for expression of osteoblast marker proteins. A representative experiment is shown. (**B**) Calcium deposition normalized to sample protein content. Solvent control = 1.00. Means + SEM, n ≤ 11. *P < 0.05, **P < 0.01, ***P < 0.001. (**C**) Representative western blot analysis for expression of osteoblast marker proteins. GAPDH serves as loading control.

**Figure 3:**
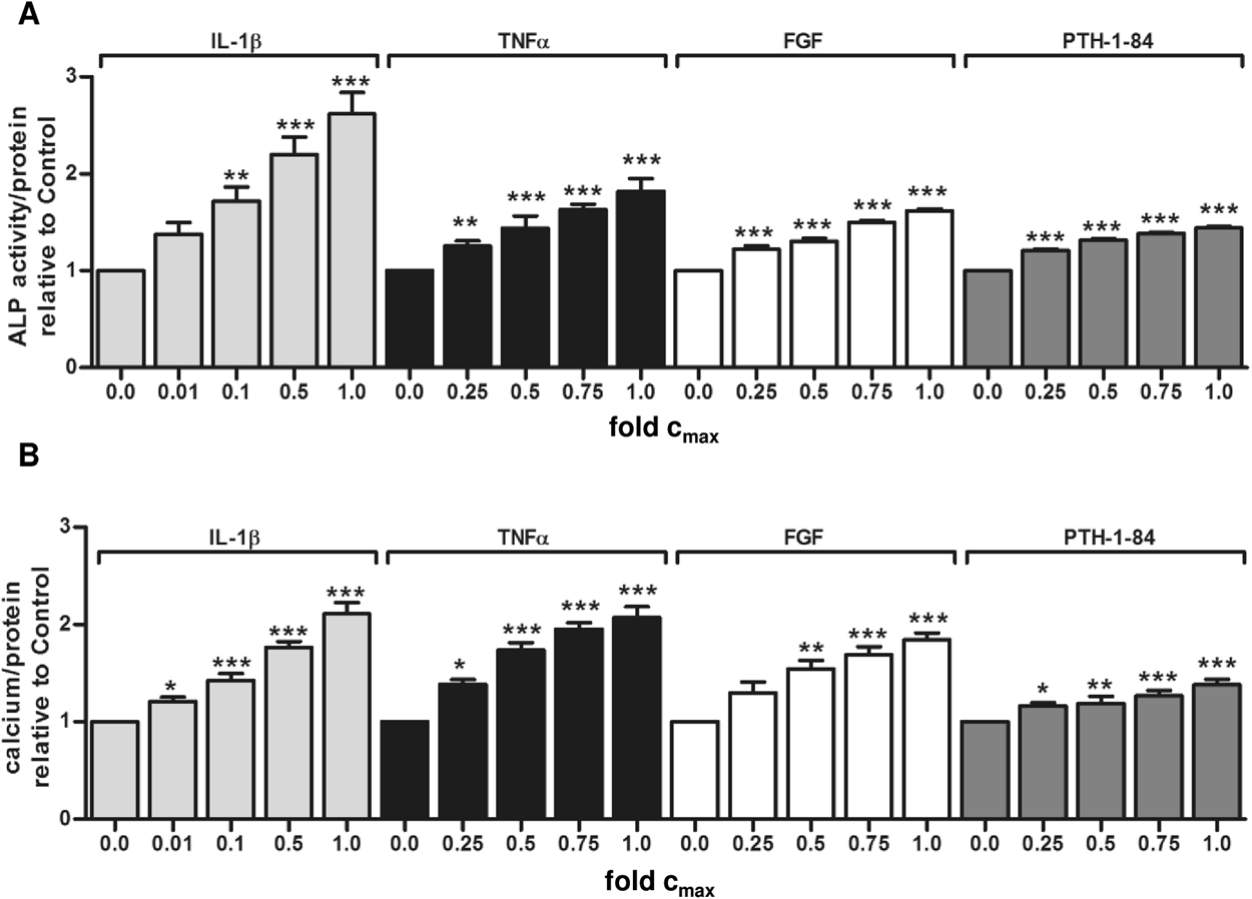
Dose-dependent induction of osteoblastic differentiation in MSC by URS. (**A**) ALP activity in MSC treated with different concentrations of toxins in OM for 7 days. (**B**) Calcium deposited by MSC cultured for 3 weeks in OM with increasing URS concentrations. “Fold c_max_” denotes the x-fold concentration of the highest concentration. Values were normalized to protein content and are expressed relative to OM without URS (set 1.00). Means + SEM, n = 4–6. *P < 0.05, **P < 0.01, ***P < 0.001.

The phenotypic changes induced by the eight uremic toxins with the strongest signal in the screening experiment were characterized in more detail. MSC exposed to the pro-inflammatory cytokines IL-1β and TNF-α produced the highest amounts of calcified extracellular matrix, which corresponds to calcification of the arterial wall, as shown by Alizarin staining (Fig. 2A) and quantitative measurements (Fig. 2B). FGF-2 and, to a lesser extent, PTH1-84 also substantially increased extracellular calcium (Fig. 2A,B). The small water soluble molecules threitol, urea, and cytidine as well as the PTH fragment PTH7-34 also increased MSC calcification (Fig. 2B) corroborating the observations of the ALP screening. Osteoblast marker protein expression studied by immunocytochemistry (Fig. 2A) and western blot analysis (Fig. 2C) including collagen I, osteopontin, osterix and Cbfa/Runx confirmed a complete phenotypic switch of MSC towards osteoblast like cells. A dose-response relationship over a broad concentration range below the EUTox c_max_^6,8^ was observed for the effects of IL-1β, TNF-α, FGF-2, and PTH1-84 on MSC osteoblast differentiation (Fig. 3A) and calcium deposition (Fig. 3B).

### Selective blockade of IL-1, TNF-α, and FGF in uremic serum prevents osteoblastic phenotype conversion of MSC

**Figure 4:**
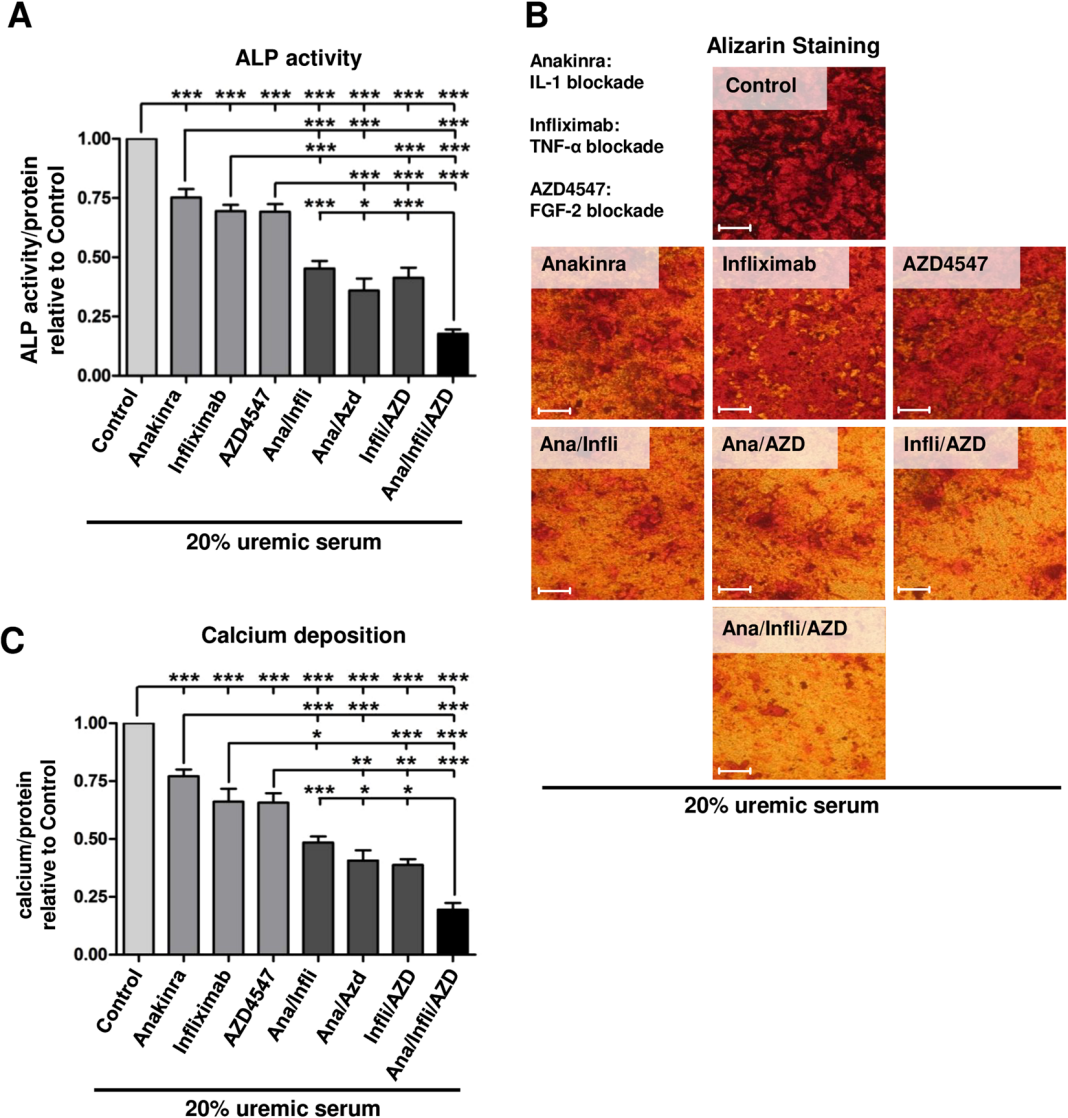
Antagonization of pro-inflammatory cytokines and fibroblast growth factor reduces osteoblast differentiation and calcification of MSC induced by serum from dialysis patients. MSC were exposed to serum from dialysis patients in OM. Inhibitors of IL-1, TNF-α, and fibroblast growth factor were added alone or in combination. (**A**) ALP activity normalized to protein content. (**B**) Alizarin staining from a representative experiment. (**C**) Deposited calcium normalized to protein content. All values are expressed relative to OM with patient serum without inhibitors (Control = 1.00). Means + SEM, n = 8. *P < 0.05, **P < 0.01, ***P < 0.001.

The identified pro-inflammatory cytokines and growth factors are part of a complex mixture of substances that accumulate in patients with end-stage renal disease. We employed specific inhibitors of either IL-1, TNF-α or FGF in a cell culture system with serum from dialysis patients to assess their relative contribution to uremia associated malfunctioning of MSC and to establish potential therapeutic targets based on their functional importance. The recombinant human IL-1 receptor antagonist (rhIL-1ra) anakinra decreased ALP activity (−25%, Fig. 4A) and levels of extracellularly deposited calcium (−23%, Fig. 4B,C) of MSC incubated with uremic serum in a dose-dependent manner (Fig S2A,B).The chimeric monoclonal antibody infliximab and the fusion protein etanercept served for TNF-α targeting. Both, infliximab (Fig. 4) and etanercept (Fig S3), reduced ALP activity (−31%, Figs 4A; −38%, and S3A) and calcium deposition (−34%, Fig. 4B,C; −31%, Fig. S3B,C) of MSC exposed to uremic serum. Again, these effects were dose-dependent (Fig. S2A,B). FGF receptor tyrosine kinase activity was blocked with the small-molecule AZD4547 which dose-dependently decreased ALP activity (−31%, Figs 4A and S2A) as well as calcium deposition (−34%, Fig. 4B,C and S2B). Combining IL-1 and TNF-α cytokine antagonists or a cytokine antagonist with the FGF receptor inhibitor was synergistic in reducing ALP activity (−46–64%, Figs 4A and S3A) and calcium deposition (−51–64%, Figs 4B,C and S3B,C). Inhibition of all three components at the same time was most effective (−70–80%, Figs 4A,C and S3A–C).

## Discussion

We embarked on a systematic approach to unravel the complex pathophysiologic effects of the uremic microenvironment on vascular integrity and regeneration that initiate and propagate uremic calcific arteriopathy responsible for the prognosis limiting high cardiovascular disease burden in CKD patients. Our work identified the pro-inflammatory cytokines IL-1β and TNF-α as the most potent enhancers of osteoblastic phenotype conversion in vascular progenitors by applying an unbiased screening strategy to test 63 individual URS on MSC. Inhibition of IL-1 and TNF-α contained in uremic serum provided interventional proof-of-concept that targeting of pro-inflammatory cytokines in a complex mixture of uremic toxins can effectively reduce osteoblastic transformation of MSC. Two other middle-sized molecules, FGF-2, and full-length PTH also induced MSC calcification, yet with lower potency. Pharmacologic blockade of a common FGF receptor alone or in combination with IL-1 and TNF-α inhibition was also beneficial with additive impact. These findings suggest a crucial role for the pro-inflammatory cytokines IL-1β and TNF-α over other middle-sized molecules in initiation of key biologic processes responsible for uremic calcifying arteriosclerosis driven by cells of the mesenchymal lineage. Targeted therapies based either on selective pharmacologic inhibition or unselective extracorporeal removal of key middle-sized molecules might translate into improved outcomes.

### Unbiased screening

Our study is the first to investigate the specific effects of a large panel of individual URS on biologic processes relevant for the development of CKD related vascular calcification by means of standardized biologic assays without hypothesis driven preselection. The capability of unfractionated uremic serum to induce an osteoblastic pro-calcific phenotype in MSC has been reported^19^, however the decisive toxins have not been identified. Following recommendations for *in vitro* testing of URS proposed by the EUTox^8^ we applied high concentrations of URS^5,6,9,20^ in the screening experiments to maximize sensitivity. The four substances with the highest potential for induction of osteoblast differentiation in MSC, IL-1β, TNF-α, FGF-2, and PTH 1–84 increased ALP activity and extracellular matrix calcification in a dose-dependent manner. As lower concentrations were also capable to induce the osteoblastic MSC phenotype, biologic relevance of our findings is broadly applicable and may extend on pre-dialytic patients as well. A potential limitation of our study is that we had to rely on systemically measured concentrations of URS although local levels in the target tissue might differ substantially. Furthermore, it remains to be determined if our findings are expandable on differentiated VSMC.

Other approaches focusing on individual URS such as guanidino compounds^21^ and certain protein bound toxins^7^ that were hypothesized to increase overall cardiovascular risk and vascular dysfunction in dialysis patients have been pursued. None of them took into consideration the variety and complexity of the uremic milieu. In our screening approach, neither guanidino compounds nor protein bound molecules substantially increased osteoblastic transformation of MSC. However, our data are concordant to a study suggesting that guanidino compounds rather inhibit calcification of VSMC^21^. Yet, several guanidino compounds are capable to stimulate production and release of pro-inflammatory cytokines including TNF-α^22^ in leukocytes that in addition accumulate due to reduced glomerular filtration rate indicative of an indirect effect on vascular calcification via sparking inflammation.

### Negative impact of pro-inflammatory cytokines on regenerative capacity of vascular progenitors

The importance of inflammation for initiation and propagation of vascular pathologies is increasingly recognized. In atherogenesis, classical metabolic stimuli such as oxidized lipids elicit a chronic inflammatory response that is part of a vicious cycle by generating more vascular damage that in turn further stimulates inflammation^23^. IL-1β and TNF-α, identified in our study as the most potent URS to enhance calcification of MSC, are hub nodes in the inflammatory network operative in coronary artery disease^24^ and have been linked to a variety of acute and chronic vascular pathologies^25^. A prototypic disease with systemic sterile inflammation characterized by elevated levels of IL-1β and TNF-α is rheumatoid arthritis (RA). Similar to CKD patients, patients with RA have an excessive risk for CVD that is beyond that of traditional risk factors^26^. Both molecules are also highly relevant in dialysis patients since they are strong predictors for mortality in this cohort in epidemiologic studies^27^. Cardiovascular complications are the leading cause of death in this population^28^ but their distribution pattern, clinical characteristics and responses to treatment differ substantially from the general population. For instance, the main vascular lesion in CKD patients is not the atherosclerotic plaque but severe calcification of the medial layer of arteries. Nevertheless, inflammation appears also to be operative in this condition evidenced by high TNF-α expression in calcified aortic tissue of patients with advanced CKD^29^. VSMC from coronary arteries have been reported to respond to TNF-α with enhanced osteoblastic differentiation and calcium deposition^30^. We now extend these findings on MSC as vascular progenitors. Osteoblastic MSC transformation induced by pro-inflammatory cytokines and other middle-sized molecules that act systemically in dialysis patients could help explain accelerated development of this therapy resistant type of generalized arteriosclerosis as a consequence of lost regenerative capacities in contrast to more localized inflammation in atherosclerotic plaques. This hypothesis is supported by an independent recent study demonstrating excessive dysfunctional matrix synthesis and disruption of tube formation by uremic serum in a three dimensional co-culture system of MSC and endothelial cells^31^. Multiple functional abnormalities in MSC related to vascular regeneration culminating in impaired *in vivo* angiogenesis and potential for ectopic osteogenesis were found in mouse models of uremia^32,33^.

### Novel therapeutic strategies

Multiple observational studies linking markers of inflammation such as high-sensitivity C-reactive protein (hsCRP) to several cardiovascular diseases^25,34^, pilot studies successfully testing IL-1 blockade in stroke^35^ and myocardial infarction^36^ as well as the cardiovascular benefits conferred by anti-inflammatory disease modifying drugs in RA including unspecific agents such as methotrexate and targeted biologicals like IL-1β and TNF-α antagonists^26,37^ led to the initiation of large randomized clinical trials testing anti-inflammatory therapies in cardiovascular conditions. For example, the Canakinumab Anti-Inflammatory Thrombosis Outcomes Study (CANTOS) enrolled more than 10,000 post-myocardial infarction patients with persistent inflammation (hsCRP > 2 mg/L). Participants were randomized to receive either placebo or different doses of the monoclonal human anti-IL-1β antibody canakinumab administered subcutaneously every three months. The hazard ratio for the primary endpoint (nonfatal myocardial infarction, nonfatal stroke, or cardiovascular death) was 0.85 (p = 0.021) for the medium dose of 150 mg in comparison to placebo. CRP levels were significantly reduced while lipid levels remained unchanged^38^. Our study adds to the body of evidence encouraging similar trials in dialysis patients, a population with even higher levels of inflammation and greater cardiovascular risk, although the predominant type of vascular lesions differs.

However, considering the severity of vascular calcification in end-stage renal disease it might not be sufficient to block only one of the multiple contributing mediators. We documented additive protective effects upon combined targeting of either IL-1, TNF-α, or FGF. Each treatment modality reduced calcium deposition and ALP activity induced by uremic serum by about 25–30%. It is tempting to speculate that multimodal therapeutic targeting would be most effective. Application of several biologic agents and small molecules or targeting common pathways downstream of pro-inflammatory cytokine and FGF signaling could offer a promising approach. For example, NF-κB activation is a common pathway of IL-1, TNF-α, and FGF-2 signal transduction^39,40^. As introduced in oncology, bi- or multispecific antibodies directed against two or more different molecules, e.g. IL-1β, TNF-α, and FGF-2, can be engineered^41^.

The “top 4” toxins identified in our screening approach, IL-1β, TNF-α, FGF-2, and PTH1-84, are “middle-sized molecules”. Conventional dialysis with high-flux dialyzers (HFL) featuring a molecular weight cut-off of 10–20 kDa fails to effectively eliminate these URS with a molecular weight of 500–60,000 Da. Maintenance dialysis with membranes characterized by a higher molecular weight cut-off (HCO) between 50 and 60 kDa resembling more closely the physiologic filtration characteristics of healthy kidneys^42^ has a greater capacity for the removal of middle-sized molecules (Fig. S4) and could positively influence serum composition to reduce its pro-osteoblastic and pro-calcifying capacities. Removal of pro-inflammatory cytokines by continuous hemofiltration with HCO dialyzers was associated with reduced norepinephrine demand in patients with sepsis^43^. Despite favorable effects on immune cell functions^44^, broader evidence for improved patient outcomes is missing. Nevertheless, advancing from dialysis with low-flux membranes to HFL membranes with larger pore size provided more effective clearance of smaller middle-sized molecules, reduced cardiac morbidity^45^, and improved survival in subgroups of incident dialysis patients^46^. Thus, it is conceivable that HCO dialysis with more effective removal of harmful mediators could translate into even better outcomes as compared to HFL dialysis. Due to significant albumin losses HCO dialysis is not feasible in the long-term. To solve this problem, more selective medium cut-off (MCO) membranes with intermediate pore sizes are being developed^47,48^. Alternatively, adsorption of middle-sized molecules including pro-inflammatory cytokines and protein-bound URS can be achieved with membranes made of novel materials^49,50^.

In a regenerative cell therapeutic approach genetically modified MSC with inactivated IL-1, TNF-α, and FGF-2 signalling could be reintroduced into patients after *ex vivo* expansion and manipulation to specifically restore vascular repair mechanisms. The advantage of this strategy would be absence of immunosuppressive side effects that may limit systemic inhibition of IL-1β and even more so of TNF-α. Yet, important obstacles such as appropriate homing had to be overcome.

In conclusion, the pro-inflammatory cytokines IL-1β and TNF-α together with other middle-sized molecules, FGF-2, and PTH1-84, act as the most potent inducers of osteoblastic phenotype conversion in MSC. Individual or simultaneous targeting of these key mediators can offset the detrimental influence of a complex uremic microenvironment on regenerative properties of MSC and effectively prevent osteoblastic transformation. Given the great unmet medical need to improve cardiovascular health in CKD and end-stage renal disease patients and the emergence of novel therapeutic paradigms such as inhibition of inflammation, an artificial kidney with a next to physiological capacity for removal of critical toxins, and cell therapy, we hope that our findings will facilitate clinical studies as the next important step.

## Conclusion

Patients with chronic kidney disease are a highest-risk population for cardiovascular morbidity and mortality due to accelerated intimal and medial calcification of arteries that cannot effectively be treated with conventional therapies such as statins.

In an unbiased approach, we identified the pro-inflammatory cytokines IL-1β and TNF-α as the key uremic triggers for calcific differentiation of MSC, progenitor cells implicated in vascular regeneration.

Pharmacologic blockade of the identified cytokines potently reduced the propensity of patient sera to enhance osteoblastic transformation in MSC.

Our results support anti-inflammatory strategies in patients with chronic kidney failure to restore vascular regenerative capacity and prevent excessive vascular damage.

## Methods

All studies involving human material were conducted in accordance with the Declaration of Helsinki and had been approved by local ethic authorities (Permission EA4/114/11, Ethikkommission Berlin; Permission 2572, Ethikkommission Ärztekammer Hamburg). All subjects provided written informed consent for isolation of bone marrow aspirates, or sample collection for uremic serum.

### Isolation and culture of MSC

To obtain sufficient numbers of vascular progenitor cells, we chose bone marrow as an easily accessible source of MSC that are regarded as counterparts of pericytes, vascular progenitors located in the vessel wall^14^. MSC were isolated from bone marrow aspirates acquired from 20 healthy, non-pregnant bone marrow donors (7 female, 13 male) median age 31 years (range 0.5–42) as described previously^51^. In brief, bone marrow mononuclear cells were purified by Ficoll density gradient centrifugation, plated at 400,000 cells/cm^2^ and cultured in α-MEM (#E15–862, PAA) supplemented with 100 U/mL penicillin (PAA), 100 μg/mL streptomycin (PAA), 2 IU/ml heparin (Ratiopharm), and 5% freshly thawed platelet lysate at 37 °C and 5% CO_2_. Nonadherent cells were washed off with PBS after 2–3 days. Medium was changed twice a week. When cultures reached about 70% confluence, cells were detached with 0.05% Trypsin/0.02% EDTA (PAA), counted, and re-plated at 500 cells/cm^2^ in 175 cm^2^ flasks (Sarstedt). For all MSC preparations, expression of characteristic surface marker proteins (CD73, CD90, CD105), and lack of hematopoetic markers as well as mesenchymal multilineage differentiation capacity were confirmed (Fig. S1) according to the standard criteria for MSC research^52^.

### Surface marker expression and multi-lineage differentiation potential

50,000 cells were labeled with 3 µl antibody or corresponding isotype control (Isotype IgG2a (BD, #553456), CD45 (BD, #555492), HLA-DR (BD, #559866), CD14 (BD, #557153), CD73 (BD, #561254), CD105 (BD, #561443), Isotype IgG2aκ (BD #555573), Isotype IgG1 (Milteny, #130-081-002), CD11b (Milteny, #130-081-201), CD34 (Milteny, #130-092-213), CD90 (Milteny, #130-095-403), CD19 (Milteny, #130-091-328)) and analyzed using a Beckton Dickinson FACS Calibur).

To induce adipogenesis, confluent monolayers were treated with DMEM containing 1 μM dexamethasone, 0.01 mg/ml insulin (Berlinchemie), 0.2 mM indomethacin (Cayman Chemical Company), and 0.5 mM 3-isobutyl-1-methyl-xanthine (Serva) for 4 weeks. After fixation with 4% paraformaldehyde, staining was performed with 6 ml of 0.5% Oil red O (Sigma) in isopropanol added to 4 ml deionized water.

Chondroblastic differentiation was tested in pellet cultures with 1 × 10^6^ cells in 4 ml DMEM containing 1 mM sodium pyruvate (Applichem), 20 mM HEPES (Roth) pH 7.3, 0.1 µM dexamethasone, 0.1 mM 2-phospho-L-ascorbic acid, and 10 ng/ml TGF-β1 (R&D). After 4 weeks, protein extraction and western blot analysis were performed.

### Induction of osteoblastic differentiation

MSC (passages 2 to 5) were seeded in complete α-MEM at 140,000 cells per well in 6-well-plates. Medium was changed the following day to osteoblast induction medium (OM) consisting of Dulbecco’s Modified Eagle’s Medium (DMEM; PAA) supplemented with 2 mM glutamine (PAA), penicillin/streptomycin (PAA), 1% FCS (PAA), 10 mM β-glycerophosphate (Applichem), 500 µM ascorbic acid, and 100 nM dexamethasone (all from Sigma).

### Panel of individual URS

The screening experiment was based on a list of URS published by the European Uremic Toxin Work Group—EUTox in 2007^8^. URS that were not available and those, which by themselves or their respective solvent exerted cytotoxic effects on MSC precluding further testing in the cell culture model, were excluded. Substances found to be elevated in uremia by members of our group were added. To screen for effects on MSC osteoblastic differentiation, individual URS were freshly added to osteoblast induction medium (OM) at the highest concentrations reported in patients with chronic renal failure requiring renal replacement therapy (C_MAX_) as suggested in the 2003 and 2007 EUTox reports^6,8^ and subsequent publications on uremic toxicity^20,53–55^. Adequate solvent controls were included in all experiments. Medium was changed every 2–3 days. Dose-response curves were generated by incubating the cells with x-fold concentrations of the respective c_max_ as indicated. Protein bound URS were applied in presence of 35 g/L human albumin as recommended by EUTox^8^. For details see Table S1.

### Uremic serum and blockade of IL-1, TNF-α, and FGF-2

To test for effects of IL-1, TNF-α, and FGF-2 contained in the blood of dialysis patients on MSC osteoblastic transformation, we used OM supplemented with 20% serum from dialysis patients instead of 1% FCS. A serum pool derived from 58 stable patients on maintenance hemodialysis (37 male, 21 female; mean age 53 ± 15 years) was used. Serum was obtained immediately prior to a dialysis session after a dialysis-free interval of 3 days. Diabetics, former transplant recipients, pregnant, and acutely ill patients were excluded. For clinical chemical analyses, see Table S2.

IL-1 was inhibited with the recombinant human IL-1 receptor antagonist (rhIL-1ra) Anakinra (Swedish Orphan Biovitrum). TNF-α was blocked either with the chimeric monoclonal antibody Infliximab (Janssen Biologics B.V.) directed against TNF-α or with the fusion protein Etanercept (Pfizer) consisting of the extracellular ligand binding domain of the TNF receptor 2 and the Fc part of human IgG1. FGF receptor tyrosine kinase activity was inhibited with the small molecule AZD4547 (Selleckchem) antagonizing FGF-2. Single agents or combinations of two or three substances were added at the indicated concentrations. Medium was changed every 2–3 days.

### Alkaline phosphatase activity

Activity of ALP in MSC was determined after exposure to the different experimental conditions for 7 days. Cells were washed with PBS and lysed with 400 µl ALP lysis buffer (150 mM Tris pH 10.0, 0.1 mM ZnCl_2_, 0.1 mM MgCl_2_, 1% Triton-X100) at room temperature under constant agitation for 30 minutes. Supernatants were collected and aliquots were immediately frozen at −80 °C. For measurement of ALP activity, an aliquot was thawed and centrifuged for 10 min at 12,000 rpm and 4 °C. Each sample was measured in triplicate. 50 µl per well of a 96-well-plate were mixed with 200 µl substrate solution (ALP buffer with freshly dissolved p-Nitrophenyl phosphate at 2.7 mM) that was pre-warmed to 37 °C. Optical densities (OD) were measured at 405 nm and followed every 10 min over a 1-h incubation period at 37 °C. ∆OD values to baseline ODs at one chosen time point during the linear phase were divided by the protein concentration of the sample as determined with the DC Protein Assay (Bio-Rad). Each ∆OD/protein ratio was related to the ∆OD/protein ratio of the appropriate control.

### Calcium deposition

Extracellular calcium deposition by differentiating MSC was assessed after 3 weeks of incubation with OM containing the indicated experimental substances. After supernatants were discarded, calcified cells were scraped off in 500 µL 0.6 M HCl, transferred to microtubes, and incubated overnight under constant agitation at 4 °C to solubilize the calcium. Samples were then centrifuged for 60 min at 20,000 g and 4 °C. Supernatants were transferred to new microtubes and pellets were dissolved in 25 µl 0.1 M NaOH/0.1% SDS solution for protein quantification with the DC protein assay (Bio-Rad). Supernatants were assayed in duplicate in 96-well-plates. 10 µL either of a calcium standard curve or sample were mixed with 150 µL color reagent (0.1 mg/mL ortho-cresophthalein complexone, 1 mg/mL 8-hydroxy-quinoline, 0.7 M HCl) and 150 µl AMP buffer (15% 2-amino-2-methyl-1-propanol in H_2_O, pH 10.7). After incubation for 15 min at room temperature, OD was measured at 540 nm. Blank absorption was subtracted and calcium concentrations were calculated by means of the standard curve. Extracellular calcium was finally expressed as µg calcium per mg protein.

### Immunohistochemistry

50,000 cells per well were seeded on 15 mm glass-coverslips (Roth) in 1% FCS/DMEM and allowed to adhere overnight. Incubation with URS was performed as described above. After 3 weeks, cells were fixed with 4% paraformaldehyde (Sigma) in PBS for 10 min, and permeabilized for 3–5 min with 0.5% Triton X-100 (Applichem) in PBS. After blocking overnight in 3% BSA/PBS at 4 °C, primary antibodies (Osteopontin abcam ab8448, Collagen I abcam ab34710; 1:500 in blocking solution) were incubated for 2 hours at 37 °C in a wet chamber. After three washes with PBS, slides were incubated with appropriate secondary antibodies (HRPO-conjugated IgG, Dianova) for 2 hours at room temperature. Signal was developed with AEC High Sensitivity Substrate Chromogen Ready to use (Dako) for about 10 minutes. Coverslips were washed extensively, and cells were counterstained with Mayer’s hematoxylin (Medite) followed by a final wash in water pH 12.6 with NaOH. Photomicrographs were taken on a Zeiss Axiovert 40 CFL using a Canon PowerShot A649.

### Alizarin staining

140,000 cells were seeded in each well of a 6 well plate in 1% FCS/DMEM. The next day, cells were stimulated as indicated. Medium was changed every 2–3 days for 3 weeks. Cells were fixed with ice-cold methanol for 30 min at −20 °C and air dried. Alizarin (1,2-dihydroxyanthraquinone, Sigma) was dissolved in 0.1 M boric acid buffer pH 4 to a final concentration of 5% and filtered. Cells were stained for 1 hour at room temperature. After several wash steps with PBS pH 6.0, wells were dried and micrographs were taken.

### Western blot

360,000 cells were differentiated in 6 cm dishes for 3 weeks in 1% FCS/OM containing the indicated substances or solvent control. Medium was changed every 2–3 days. Cells were lysed (20 mM Tris pH 7.5, 350 mM NaCl, 1% Triton X-100) for 20 min on ice. After centrifugation, protein concentrations in the supernatants were quantified with the DC protein assay (Bio-Rad). 5x Laemmli buffer (250 mM Tris pH 6.8, 500 mM DTT, 10% SDS, 0.5% Bromophenol blue, 35% Glycerol) was added, and samples were heated to 99 °C for 5 min. 50 µg total protein per lane were separated by SDS-polyacrylamid-gel-electrophoresis and electrotransferred to a PVDF membrane (GE Healthcare) following standard protocols. Blocking was performed with 10% BSA/TBS-T for 2 hours at room temperature. All antibodies were diluted in blocking solution: Osterix (abcam), Cbfa/Runx (MBL), Fibronectin (Santa Cruz), Collagen I (abcam) and Collagen IIa1 (Santa Cruz) 1:500; Osteopontin (abcam) 1:1000. GAPDH (hytest) served as a loading control and was applied at 1:100,000. After incubation with appropriate secondary antibodies (Dianova), SuperSignal West Pico Chemiluminescent Substrate (Thermo Fisher) was used for development in a G:BOX F3 device (Syngene).

### Statistics

All data are expressed as mean + SEM. The screening experiments were evaluated with the Wilcoxon signed-rank test or, after confirming normal distribution of the data with the Kolmogorov-Smirnov test, with the t-test. 1-way ANOVA followed by Dunnett’s post-test was used to evaluate dose-response curves. Cytokine and FGF inhibitors were tested with 1-way ANOVA and Bonferroni’s multiple comparison test. All analyses were performed with GraphPad Prism version 5.02 for Windows, GraphPad Software, San Diego California USA. Significance was considered at a value of p < 0.05.

## Electronic supplementary material

Supplementary Dataset 1

## Data Availability

The datasets generated during and/or analyzed during the current study are available from the corresponding author on reasonable request.
