# Supplementary material for: Targeting proinflammatory cytokines ameliorates calcifying phenotype conversion of vascular progenitors under uremic conditions in vitro
Source: Sci Rep. 2018 Aug 14;8:12087. doi: 10.1038/s41598-018-30626-z (PMC6092400; doi:10.1038/s41598-018-30626-z)

**Supplementary Information: Tables, Supplementary Figures, Legends, and Full blot images**

**Targeting proinflammatory cytokines ameliorates calcifying phenotype conversion of vascular progenitors under uremic conditions in vitro**

Björn Hegner; Theres Schaub; Daniel Janke; Daniel Zickler; Claudia Lange; Matthias Girndt; Joachim Jankowski; Ralf Schindler; Duska Dragun

**Supplementary Table S1: Concentration and solvents of applied uremic toxins**

| **Solute** | **c_max_ [/L]** | | **Solvent** | **Supplier** | **Reference** |
| --- | --- | --- | --- | --- | --- |
| 1-Methyladenosine | 216,40 | µg | 0.9% NaCl | Sigma-Aldrich | [1](#_ENREF_1) |
| 2-Methoxyresorcinol | 322,00 | µg | 20% Ethanol | Synthesis Jankowski | [1](#_ENREF_1) |
| α-N-Acetylarginine | 4,58 | mg | 0.9% NaCl | Sigma-Aldrich | [1](#_ENREF_1) |
| Angioprotectin | 22,00 | ng | 0.1% BSA/ddH_2_O | Synthesis Jankowski | [2](#_ENREF_2) |
| Angiotensin A | 50,40 | ng | 0.1% BSA/ddH_2_O | Synthesis Jankowski | [3](#_ENREF_3) |
| Angiotensin II | 186,30 | ng | 0.1% BSA/ddH_2_O | Sigma-Aldrich | [3](#_ENREF_3) |
| Arabitol | 33,00 | mg | 0.9% NaCl | Sigma-Aldrich | [1](#_ENREF_1) |
| Asymetric dimethylarginine.2HCl | 9,94 | mg | 0.9% NaCl | Merck-Millipore | [1](#_ENREF_1) |
| Benzylalcohol | 187,90 | mg | 0.9% NaCl | Sigma-Aldrich | [1](#_ENREF_1) |
| β-Guanidinopropionic acid | 65,40 | µg | 0.9% NaCl | Sigma-Aldrich | [1](#_ENREF_1) |
| Connective tissue growth factor | 100,00 | µg | NaAcetat | Immunotools | [1](#_ENREF_1) |
| Creatine.monohydrate | 268,20 | mg | 0.9% NaCl | Merck-Millipore | [1](#_ENREF_1) |
| Creatinine | 240,00 | mg | 0.9% NaCl | Merck-Millipore | [1](#_ENREF_1) |
| Cytidine | 1,26 | mg | 0.9% NaCl | Sigma-Aldrich | [1](#_ENREF_1) |
| Dimethylglycine | 1,04 | mg | 0.9% NaCl | Sigma-Aldrich | [1](#_ENREF_1) |
| Endothelin-1 | 129,40 | ng | ddH_2_O | Sigma-Aldrich | [1](#_ENREF_1) |
| Erythritol | 34,00 | mg | 0.9% NaCl | Sigma-Aldrich | [1](#_ENREF_1) |
| Fibroblast growth factor-2 | 19,50 | ng | 0.1% BSA/ddH_2_O | Immunotools | [1](#_ENREF_1) |
| γ-Guanidinobutyric acid | 1,75 | mg | 0.9% NaCl | Sigma-Aldrich | [1](#_ENREF_1) |
| Guanidine.HCl | 1,29 | mg | 0.9% NaCl | Sigma-Aldrich | [1](#_ENREF_1) |
| Guanidinoacetic acid | 694,00 | µg | 0.9% NaCl | Sigma-Aldrich | [1](#_ENREF_1) |
| Hippuric acid | 471,00 | mg | 0.25 M NaOH/Tris | Sigma-Aldrich | [1](#_ENREF_1) |
| Hydroquinone | 286,00 | µg | 0.9% NaCl | Sigma-Aldrich | [1](#_ENREF_1) |
| Hypoxanthine | 5,30 | mg | 0.25 M NaOH/Tris | Sigma-Aldrich | [1](#_ENREF_1) |
| Indole-3-acetic acid | 9,08 | mg | 20% Ethanol | Sigma-Aldrich | [1](#_ENREF_1) |
| Indoxyl sulfate potassium salt | 279,50 | mg | 0.9% NaCl | Sigma-Aldrich | [1](#_ENREF_1) |
| Interleukin-1β | 1,70 | µg | 0.1% BSA/ddH_2_O | Immunotools | [1](#_ENREF_1) |
| Interleukin-6 | 328,10 | ng | 0.1% BSA/ddH_2_O | Immunotools | [1](#_ENREF_1) |
| Interleukin-10 | 22,60 | µg | 0.1% BSA/ddH_2_O | Immunotools | [4](#_ENREF_4) |
| Interleukin-18 | 1,14 | µg | 0.1% BSA/ddH_2_O | MBL | [5](#_ENREF_5) |
| Kynurenic acid | 9,50 | mg | 0.1 M NaOH | Sigma-Aldrich | [1](#_ENREF_1) |
| Kynurenine | 952,60 | µg | 0.9% NaCl | Sigma-Aldrich | [1](#_ENREF_1) |
| Leptin | 490,00 | µg | ddH_2_O | Immunotools | [1](#_ENREF_1) |
| Malondialdehydetetrabutylammonium | 3,35 | mg | 0.9% NaCl | Sigma-Aldrich | [1](#_ENREF_1) |
| Mannitol | 76,00 | mg | 0.9% NaCl | Sigma-Aldrich | [1](#_ENREF_1) |
| Melatonin | 436,20 | ng | 20% Ethanol | Sigma-Aldrich | [1](#_ENREF_1) |
| Methylguanidine.HCl | 2,73 | mg | 0.9% NaCl | Sigma-Aldrich | [1](#_ENREF_1) |
| Myoinositol | 232,00 | mg | 0.9% NaCl | Sigma-Aldrich | [1](#_ENREF_1) |
| N^2^,N^2^-dimethylguanosine | 415,80 | µg | DMSO | Sigma-Aldrich | [1](#_ENREF_1) |
| N^4^-acetylcytidine | 221,20 | µg | 0.9% NaCl | Sigma-Aldrich | [1](#_ENREF_1) |
| Orotic acid | 38,70 | mg | 0.1 M NaOH | Sigma-Aldrich | [1](#_ENREF_1) |
| Oxalate | 7,60 | mg | 0.9% NaCl | Sigma-Aldrich | [1](#_ENREF_1) |
| Parathormon 1-84 | 2,40 | µg | 0.1% BSA/ddH_2_O | MBS | [1](#_ENREF_1) |
| Parathormon 7-34 | 885,00 | ng | 0.1% BSA/ddH_2_O | MBS | [1](#_ENREF_1) |
| p-Cresylsulphate | 41,00 | mg | MeOH | Synthesis Jankowski | [4](#_ENREF_4) |
| Phenol | 10,50 | mg | 0.9% NaCl | Sigma-Aldrich | [1](#_ENREF_1) |
| p-OH Hippuric acid | 31,50 | mg | MeOH | Bachem | [1](#_ENREF_1) |
| Putrescine | 132,00 | µg | 0.9% NaCl | Sigma-Aldrich | [1](#_ENREF_1) |
| Quinolinic acid | 3,30 | mg | 0.9% NaCl | Sigma-Aldrich | [1](#_ENREF_1) |
| Resistin | 62,20 | µg | 0.1% BSA/ddH_2_O | Immunotools | [4](#_ENREF_4) |
| Sorbitol | 7,30 | mg | 0.9% NaCl | Sigma-Aldrich | [1](#_ENREF_1) |
| Spermidine | 187,20 | µg | 0.9% NaCl | Sigma-Aldrich | [1](#_ENREF_1) |
| Spermine | 66,70 | µg | 0.9% NaCl | Sigma-Aldrich | [1](#_ENREF_1) |
| Symmetric dimethylarginine | 1,23 | mg | 0.9% NaCl | Sigma-Aldrich | [1](#_ENREF_1) |
| Threitol | 5,70 | mg | 0.9% NaCl | Sigma-Aldrich | [1](#_ENREF_1) |
| Thymine | 11,20 | mg | 0.2 M NaOH/Tris | Sigma-Aldrich | [1](#_ENREF_1) |
| Tumor necrosis factor-α | 408,00 | ng | 0.1% BSA/ddH_2_O | Immunotools | [1](#_ENREF_1) |
| Uracil | 448,00 | µg | 0.2 M NaOH/Tris | Sigma-Aldrich | [1](#_ENREF_1) |
| Urea | 4,60 | g | 0.9% NaCl | Sigma-Aldrich | [1](#_ENREF_1) |
| Uric acid | 146,70 | mg | 0.25 M NaOH/Tris | Sigma-Aldrich | [1](#_ENREF_1) |
| Uridine | 32,60 | mg | 0.2 M NaOH/Tris | Sigma-Aldrich | [1](#_ENREF_1) |
| Xanthine | 3,44 | mg | 0.2 M NaOH/Tris | Sigma-Aldrich | [1](#_ENREF_1) |
| Xanthosine | 222,40 | µg | 0.1 M NaOH | Sigma-Aldrich | [1](#_ENREF_1) |
|  |  |  |  |  |  |

The highest concentrations reported in patients requiring chronic renal replacement therapy (c_max_) according to the European Uremic Toxin Work Group (EUTox) reports)[^1^](#_ENREF_1)^,^[^6^](#_ENREF_6) and subsequent publications on uremic toxicity[^2-5^](#_ENREF_2) were applied. Adequate solvent controls were included. Protein bound toxins were applied in presence of 35 g/L human albumin.

1 Cohen, G. *et al.* Review on uraemic toxins III: recommendations for handling uraemic retention solutes in vitro--towards a standardized approach for research on uraemia. *Nephrol Dial Transplant* **22**, 3381-3390, doi:gfm210 [pii]10.1093/ndt/gfm210 (2007).

2 Jankowski, V. *et al.* Angioprotectin: an angiotensin II-like peptide causing vasodilatory effects. *FASEB J* **25**, 2987-2995, doi:fj.11-185470 [pii]10.1096/fj.11-185470 (2011).

3 Jankowski, V. *et al.* Mass-spectrometric identification of a novel angiotensin peptide in human plasma. *Arterioscler Thromb Vasc Biol* **27**, 297-302, doi:01.ATV.0000253889.09765.5f [pii]10.1161/01.ATV.0000253889.09765.5f (2007).

4 Duranton, F. *et al.* Normal and pathologic concentrations of uremic toxins. *J Am Soc Nephrol* **23**, 1258-1270, doi:ASN.2011121175 [pii]10.1681/ASN.2011121175 (2012).

5 Meert, N. *et al.* Inconsistency of reported uremic toxin concentrations. *Artif Organs* **31**, 600-611, doi:AOR434 [pii]10.1111/j.1525-1594.2007.00434.x (2007).

6 Vanholder, R. *et al.* Review on uremic toxins: classification, concentration, and interindividual variability. *Kidney Int* **63**, 1934-1943, doi:kid924 [pii]10.1046/j.1523-1755.2003.00924.x (2003).

**Supplementary Table S2: Clinical chemical analyses**

|  |  | Uremic | Reference range |
| --- | --- | --- | --- |
| Calcium | mmol/L | 2.26 | 2.25-2.75 |
| Phosphate | mmol/L | 1.90 | 1.15-2.15 |
| Creatinine | mg/dL | 9.71 | <1-1.2 |
| Urea | mg/dL | 137 | 17-43 |
| Protein | g/L | 64.3 | 51-73 |
| Albumin | g/L | 39.7 | 38-54 |
| CRP | mg/L | 10.2 | <5 |
| Parathyroid hormone | ng/L | 262.1 | 14.9-56.9 |
| 25-Hydroxyvitamin D | nmol/L | 91.3 | 75-175 |
| 1,25-Dihydroxyvitamin D | pmol/L | 15.0 | 39-193 |

Measurements were performed by the core facility “Labor Berlin” (accreditation according to DIN EN ISO 15189:2014 under the number D-ML-134403-03) according to standard protocols.

**Supplementary Figures**


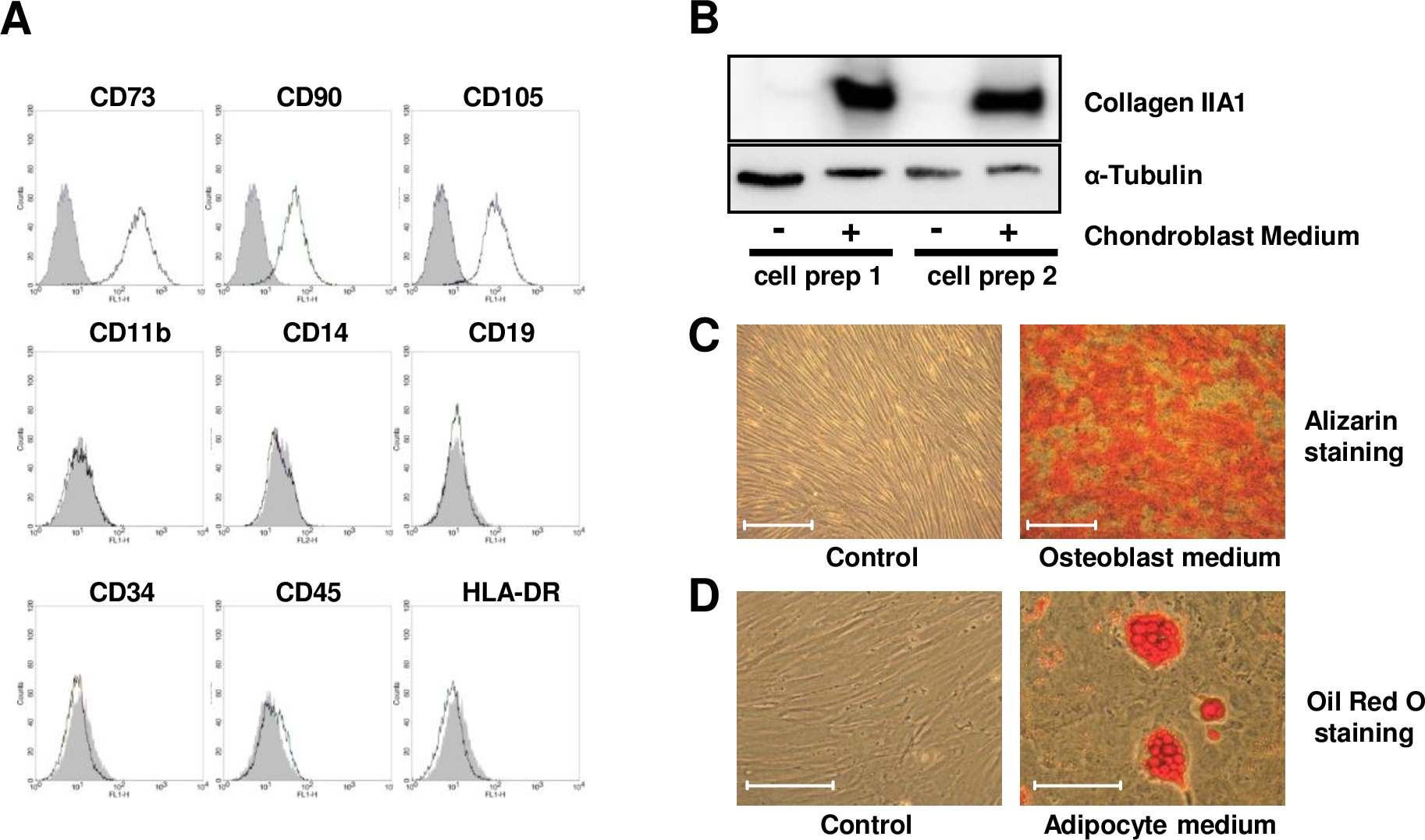


**Supplementary Figure S1:** **Analysis of cell surface marker expression and multilineage differentiation potential of plastic adherent human bone-marrow derived MSC**.

(A) Representative FACS analysis of an MSC defining surface marker panel confirming homogeneity of isolated cells. More than 98% of cells are positive for CD73, CD90, and CD105 and negative for CD11b, CD14, CD19, CD34, CD45, and HLA-DR compared to isotype control (grey). (B) Chondroblastic differentiation of MSC after incubation for 32 days with chondroblast induction medium shown by western blot analysis for chondrocyte specific type IIa1 collagen. Two representative cell preparations are shown. (C) Osteoblastic differentiation of MSC after incubation for 21 days with osteoblast induction medium shown by staining of extracellular hydroxyapatite deposits with Alizarin red S. One representative experiment is shown. Phase contrast microscopy, original magnification x100, scale bars = 50 µm. (D) Adipocytic differentiation of MSC after incubation for 21 days with adipocyte induction medium shown by staining of intracellular lipid droplets with Oil red O. One representative experiment is shown. Phase contrast microscopy, original magnification x200, scale bars = 50 µm.

**Supplementary Figure S2:** **Dose dependent reduction of osteoblast differentiation and calcification induced by serum from dialysis patients in MSC with inhibitors of pro-inflammatory cytokines and fibroblast growth factor.**


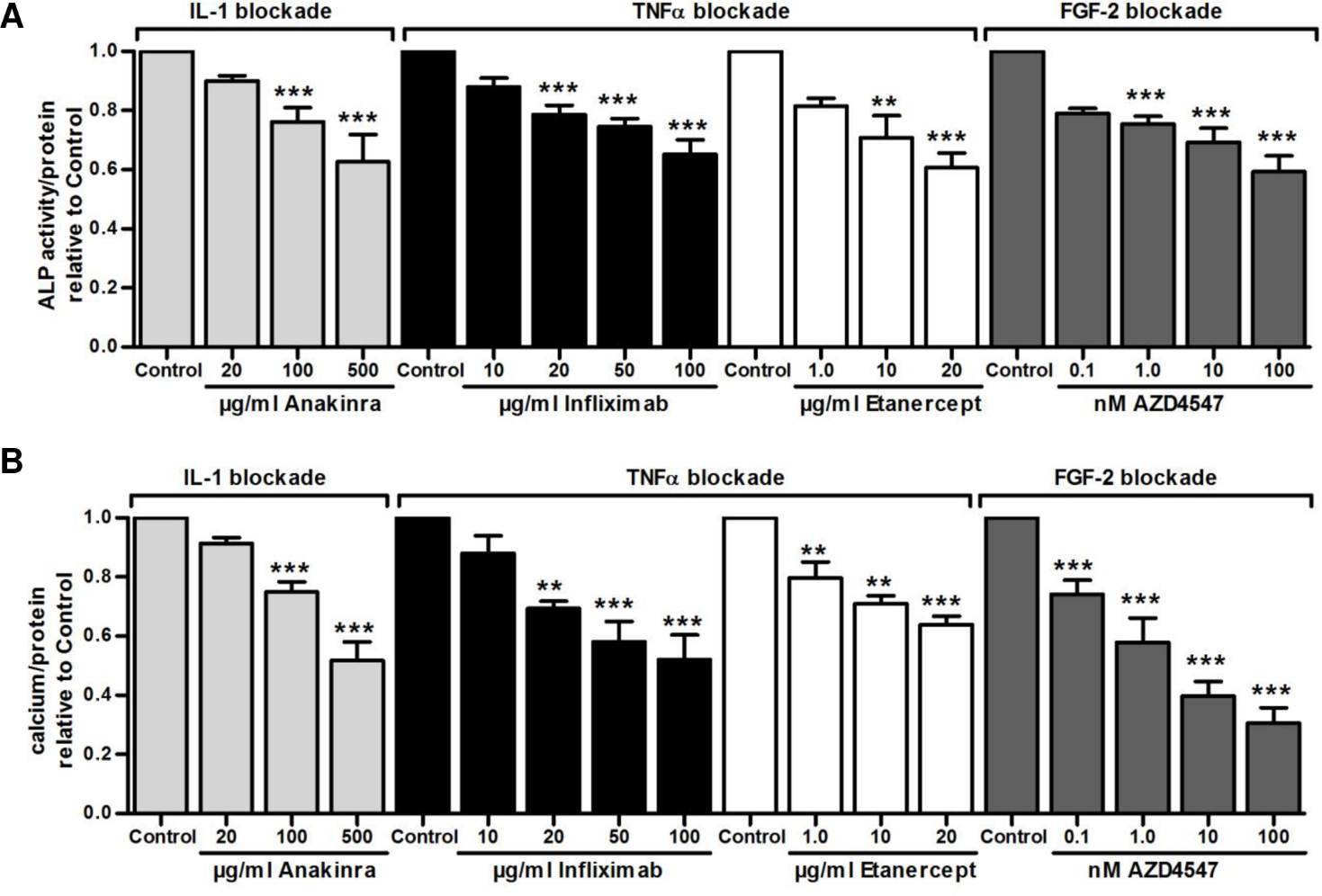


MSC were incubated with serum from dialysis patients in osteoblast induction medium (OM). Inhibitors of IL-1 (Anakinra), TNF-α (Infliximab; Etanercept), and fibroblast growth factor (AZD4547) were added at the indicated concentrations to medium containing 20% uremic serum. (A) Alkaline phosphatase (ALP) activity and (B) deposited calcium were normalized to sample protein content. All values are expressed relative to OM with patient serum without inhibitors (Control = 1.00). Means+SEM, n=8. **P<0.01, ***P<0.001.


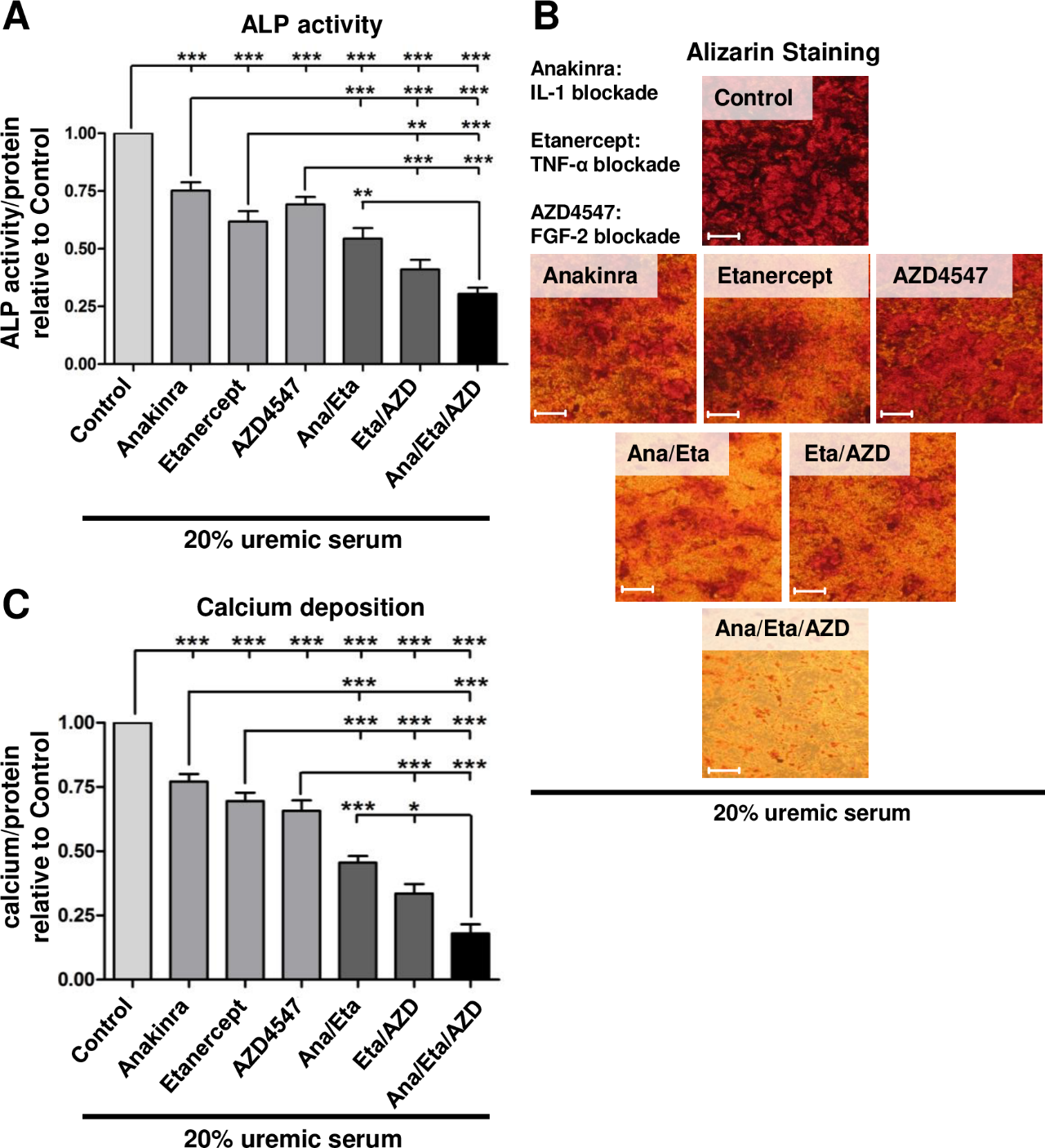


**Supplementary Figure S3:** **Effect of the TNF-α blocker Etanercept on osteoblast differentiation and calcification induced by serum from dialysis patients in mesenchymal stromal cells (MSC).**

MSC were exposed to serum from dialysis patients in osteoblast induction medium (OM). Inhibitors of IL-1 (Anakinra, Ana; 100 µg/mL), TNF-α (Etanercept, Eta; 20 µg/mL), and fibroblast growth factor (AZD4547, AZD; 10 nM) were added alone or in combination. (A) Alkaline phosphatase (ALP) activity normalized to sample protein content. (B) Alizarin red S staining of a representative experiment. (C) Deposited calcium normalized to sample protein content. All values are expressed relative to OM with patient serum without inhibitors (Control = 1.00). Means + SEM, n=8. *P<0.05, **P<0.01, ***P<0.001.


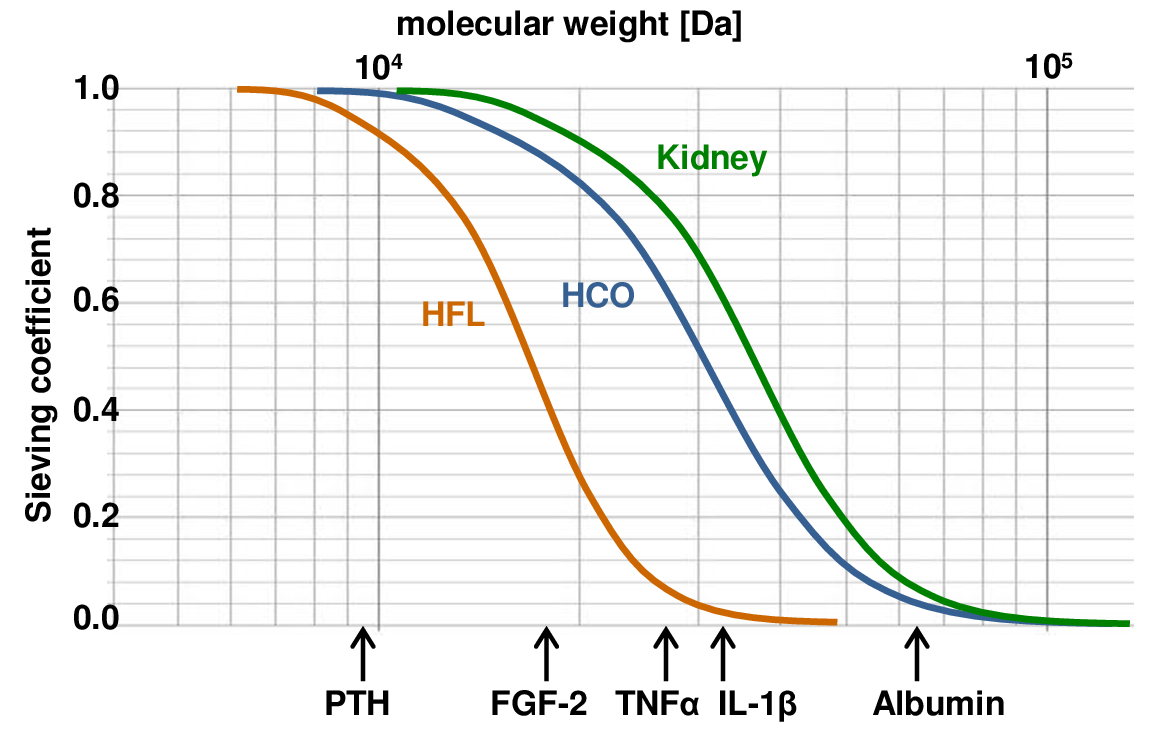


**Supplementary Figure S4: Elimination characteristics for selected middle-sized molecules and albumin of conventional HFL membranes, HCO membranes, and the kidney.**

**Full Blot Images**

**Figure 2 anti Collagen I detection**


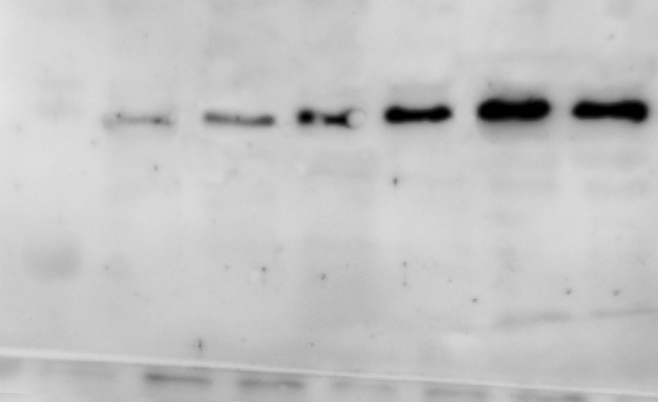


**Figure 2 anti Osteopontin detection**


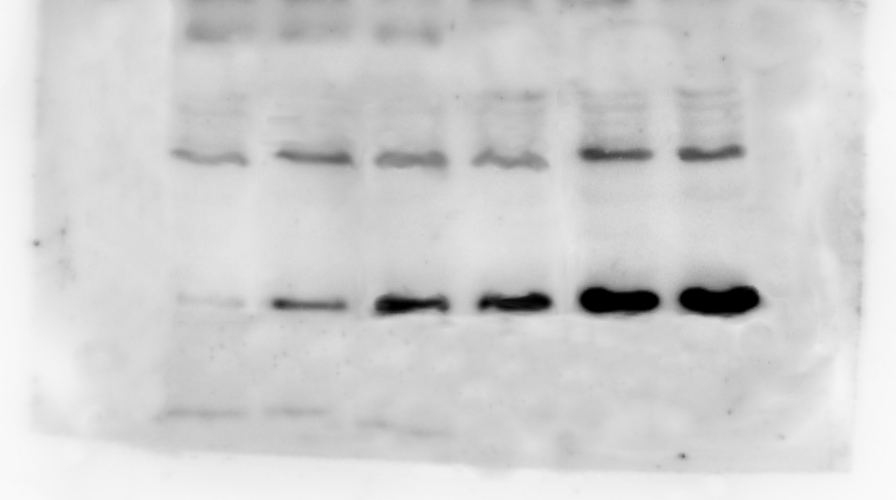


**Figure 2 anti Osterix detection**


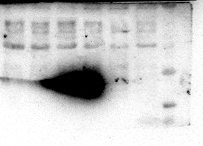


**Figure 2 anti GAPDH detection**


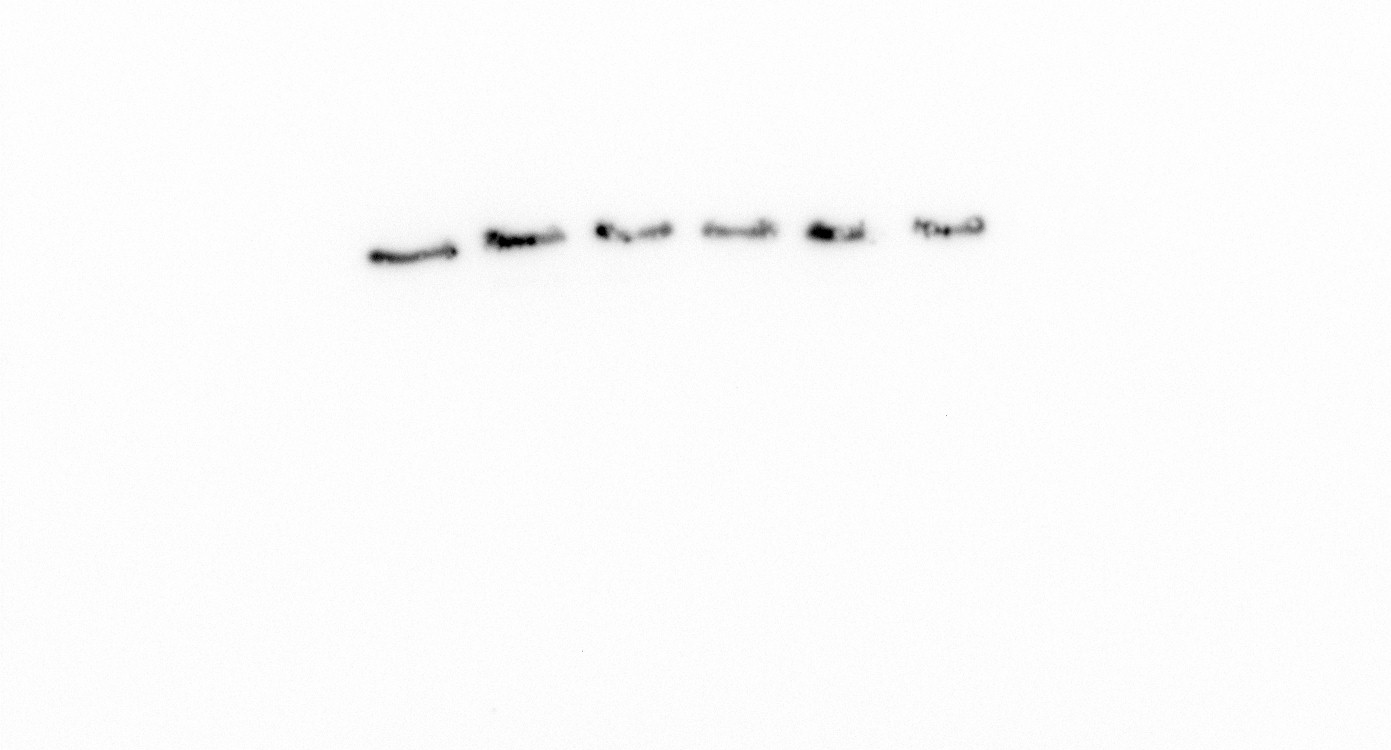


**Figure 2 anti Cbfa detection**


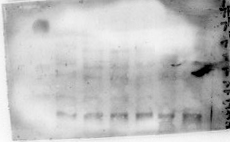


**Supplement Figure 1 anti Collagen 2A detection**


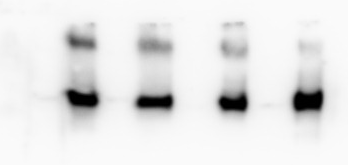


**Supplement Figure 1 anti Tubulin detection**


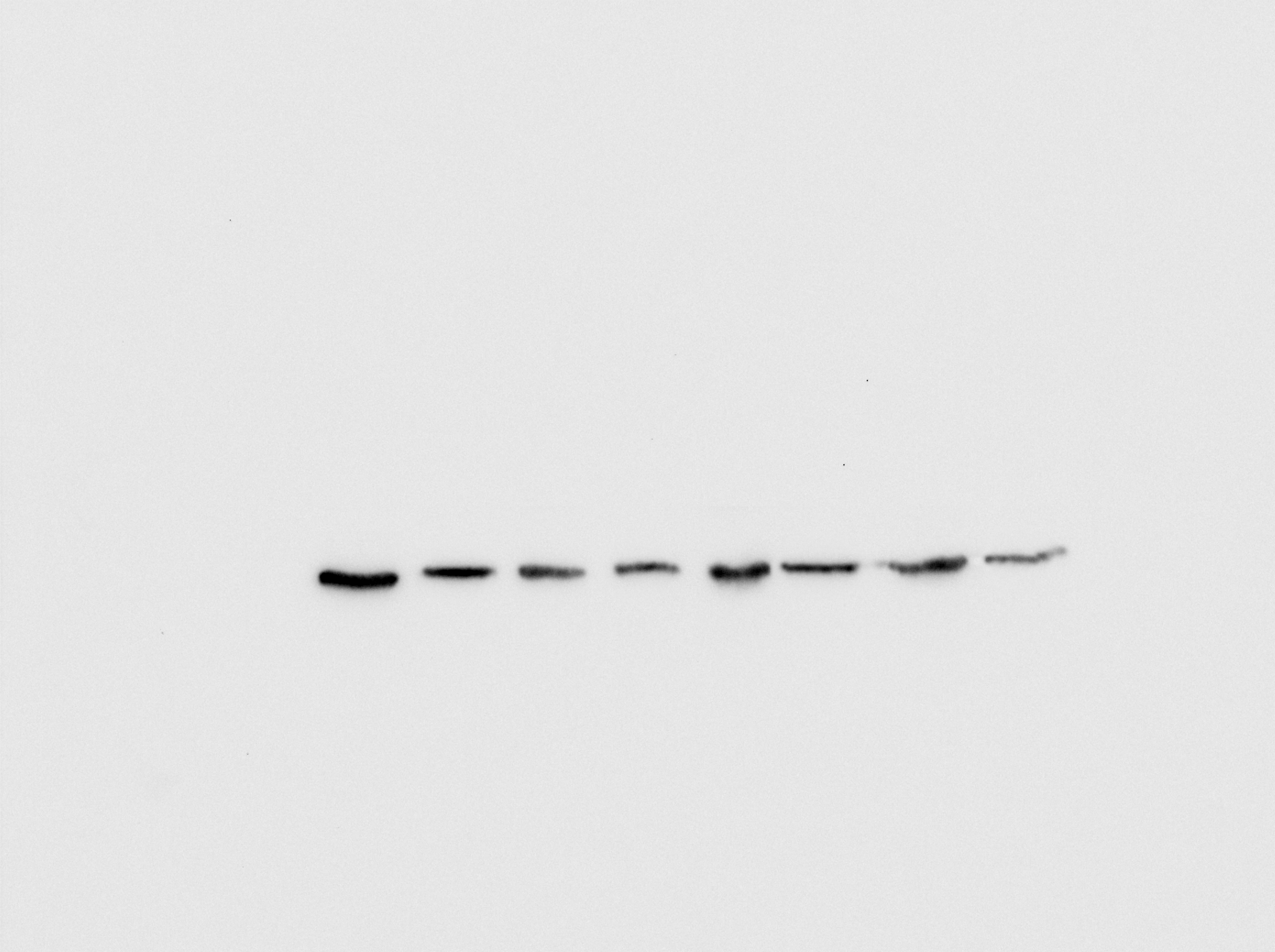

Supplement: Supplementary file 1 — Supplementary Dataset 1 [file 41598_2018_30626_MOESM1_ESM.docx]
